# Relevance of Non-communicable Comorbidities for the Development of the Severe Forms of Dengue: A Systematic Literature Review

**DOI:** 10.1371/journal.pntd.0004284

**Published:** 2016-01-04

**Authors:** Joao Toledo, Leyanna George, Eric Martinez, Adhara Lazaro, Wai Wai Han, Giovanini E. Coelho, Silvia Runge Ranzinger, Olaf Horstick

**Affiliations:** 1 Consultant in Public Health, Ministry of Health, Brasilia, Brazil; 2 Department of Community Medicine, Amrita School of Medicine, Amrita Vishwa Vidyapeetham University, Kochi, India; 3 Instituto Pedro Kouri, La Habana, Cuba; 4 Consultant in Public Health, Institute of Public Health, University of Heidelberg, Heidelberg, Germany; 5 Ministry of Health, Rangoon, Myanmar; 6 Coordinator of the Brazilian Dengue Programme, Ministry of Health, Brasilia, Brazil; 7 Consultant in Public Health, Ludwigsburg, Institute of Public Health, University of Heidelberg, Heidelberg, Germany; 8 Institute of Public Health, University of Heidelberg, Heidelberg, Germany; Centers for Disease Control and Prevention, UNITED STATES

## Abstract

Patients with dengue fever and comorbidities seem to be at higher risk of developing complications and/or severe dengue compared to healthier individuals. This study systematically reviews the evidence related to comorbidities and dengue. A systematic literature review was performed in five databases (EMBASE, PUBMED, Global Health, SciELO, Cochrane) and grey literature for full-text articles since its inceptions until October 10, 2015. A total of 230 articles were retrieved. Sixteen studies were analysed after applying all inclusion and exclusion criteria. Seven case control studies and nine retrospective cohort studies showed that comorbidities may contribute to severe dengue, especially 1) cardiovascular disease, 2) stroke, 3) diabetes, 4) respiratory disease and 5) renal disease, as well as old age. However, due to heterogeneity in studies, the real estimate effect of comorbidities as modifiers of dengue severity could not be established. Further research in regions with high prevalence of dengue infection would contribute to a better understanding of the relevance of comorbidities in severe dengue, especially with a standardised protocol, for outcomes, specific comorbidities, study design—best using prospective designs—and sample sizes.

## Author Summary

Dengue fever is a viral disease, transmitted by *Aedes* mosquitoes. Although for most cases of dengue fever the illness is self-limiting or asymptomatic, severe dengue can occur. Severe dengue is now classified by 1) plasma leakage, and/or 2) severe haemorrhage and/or 3) organ failure. Complications and deaths occur in this group of cases with severe dengue. Patients with dengue fever and comorbidities seem to be at higher risk of developing complications and/or severe dengue compared to healthier individuals. This study systematically reviews the evidence related to comorbidities and the severe forms of dengue fever. Sixteen studies were analysed after applying all inclusion and exclusion criteria. Seven case control studies and nine retrospective cohort studies assessed comorbidities and development of severe forms of dengue. The results showed that comorbidities are relevant to severe dengue, especially 1) cardiovascular disease, 2) stroke, 3) diabetes, 4) respiratory disease and 5) renal disease, as well as old age. The study of comorbidities in dengue fever is fundamental for improved patient outcome by differential case management of patients, reducing the burden of the disease. Further research in regions with high prevalence of dengue infection would contribute to a better understanding of the relevance of comorbidities with severe forms of dengue fever. An agreed protocol for such studies is urgently needed.

## Introduction

Dengue fever is an acute systemic infectious disease affecting mainly people in tropical and subtropical regions [1]. Dengue virus (*Flaviviridae* family, *Flavivirus* genus) has four different subtypes (DENV-1, DENV-2, DENV-3, DENV-4) and it is transmitted by infected *Aedes spp* mosquitoes *(Aedes aegypti* and *albopictus*) [2, 3, 4] According to estimates of Bhatt et al [5], there are around 390 million dengue fever infections per year.

There is no specific treatment for dengue fever [2, 3, 4, 6]. The gold standard therapy of clinical management of severe cases of dengue—mostly related to plasma leakage or severe bleeding, but also to organ failure—is fluid replacement, both orally and intravenously (crystalloids and/or colloids) [7]. Intensive care monitoring and assessment of plasma leakage are vital. As the pathophysiology of dengue fever involves an increase in vascular permeability, rapid fluid replacement is needed—however, fluid replacement in excess might lead to hypervolaemia, pulmonary oedema and respiratory distress. This is an aspect to be carefully evaluated and observed especially in elderly patients, due to their reduction in cardiovascular output, pulmonary compliance and renal output, resulting in clinical complications [3, 4, 6, 7].

During the 1950’s and 1960’s, the majority of dengue cases were described in children, in South East Asian countries [8]. Introduction of the transmitting vector in wild environments and urban areas, human migration and the presence of artificial egg reservoirs (described for example in used tires) have facilitated the spread the disease in many different regions, from the Caribbean islands to Brazil and from the Pacific islands to other South Asian countries causing major epidemics [2, 6, 9, 10]. From a disease initially affecting children and young adults, dengue began to affect older people [2, 7]. At the same time, many of these countries and regions where dengue is highly prevalent began to face the phenomenon of epidemiological transition [11]. The niche occupied by communicable diseases in the overall mortality rate has given space to non-communicable diseases, such as hypertension, diabetes and malignancies, with the ageing of population. As a result, dengue now affects older adults, an age group with inherently more comorbidities.

Some authors have postulated that in adults, non-communicable comorbidities and other underlying medical conditions may have a role in predisposing individuals to the severe forms of dengue [7, 12]. These comorbidities include cardiovascular diseases, endocrine diseases, allergies, haematological diseases, chronic hepatopathy, recipients of solid organ transplant, chronic renal insufficiency, autoimmune disorders, and also the condition old age [7].

Thus, the understanding of the relevance of comorbidities in the development of severe dengue is fundamental in order to better target clinical monitoring and interventions for improved clinical outcome.

The objective of this study is to systematically review the existing literature on the relevance of non-communicable comorbidities, such as hypertension, diabetes mellitus, allergies, and also old age, for the development of severe dengue and to evaluate the association between these specific comorbidities and the severity of clinical dengue expression. The scope of this review is intentionally very broad, to highlight the importance of comorbidities in relation to dengue and to stimulate a discussion about further research and its directions.

## Methods

The systematic review was performed in six databases (Cochrane Library, EMBASE, Global Health, MEDLINE, SciELO and Google Scholar) from their inceptions until October 10, 2015. Reference lists and grey literature were also searched for relevant articles.

Using the PICO format (acronym for “population or problem”, intervention or exposure of interest”, “comparison” and “outcome”) [13], the research question was framed as: “is the severity of dengue influenced by comorbidities?”.

The categories for the search included: (a) Population: adults aged 15 years or older; (b) Intervention or Exposure: dengue and comorbidities; (c) Comparison: comparison of dengue severity in individuals with and without comorbidities (this included the terms dengue fever (DF), dengue haemorrhagic fever (DHF) and dengue shock syndrome (DSS) of the 1997 WHO case classification and severe dengue (SD), for the 2009 WHO dengue case classification); (d) Outcomes: mortality and length of hospital stay.

MeSH terms, Boolean operators AND and OR and truncation ($) were inserted in the OvidSP platform (*EMBASE*, *MEDLINE* and *Global Health* articles), the *Cochrane Librar*y and in the *SciELO* database (Table 1 for the search terms). Because the *SciELO* database has a simplified search interface, the only keywords used with that database were DENGUE and COMORBIDIT*.

**Table 1:** Keywords for the search related terms and synonyms.

Keyword	Related terms and synonyms
Adult	Adult / “80 and over” / aged / elderly / “frail elderly” / “middle aged” / “young adult”
Dengue	Dengue / “dengue fever” / “dengue haemorraghic fever” / “dengue shock syndrome”
Comorbidit*	comorbidit* / allerg* / asthma* / “chronic hepatitis” OR hepatopathy / “chronic obstructive pulmonary disease” OR COPD / corticosteroid / “diabetes mellitus” OR diabetes / hyperlipidemia OR dislipidemia / hypertension / malignanc* / “sickle cell an$emia” OR “sickle cell disease” / stroke OR “heart disease / transplant* / uremia OR “chronic uremia” OR / “uremia syndrome” OR “renal failure”
Rate	death/fatality OR “fatality rate” /incidence OR “incidence rate”/ “length of stay”/ mortality OR “mortality rate”

The process to identify potentially eligible studies was based on the *Preferred Reporting Items for Systematic Reviews and Meta-Analyses* (PRISMA) flowchart [14], comprising four steps: (a) identification, (b) screening, (c) eligibility and (d) inclusion of studies. The articles obtained from that search were exported to the software *EndNote X6*.*0*.*1 (Bld 8432*, *Thomson Reuters)*. An additional search of records identified through grey literature was performed and included in the set. All duplicates were excluded through *EndNote* and manually as well. Then, articles that did not mention the word dengue or its variations in neither the title nor the abstract were excluded from the remaining articles. The remaining pool of articles was carefully reviewed to identify those that might be relevant to the question.

Inclusion criteria for studies in this review were: clinical studies in humans, studies about dengue fever and non-communicable comorbidities, adults aged > 15 years old, any of the outcomes: death, fatality rate, mortality and length of hospital stay, cohort studies and case-control studies and studies in English, French, Portuguese and Spanish.

Articles were excluded based on the following exclusion criteria: non clinical studies, non-communicable comorbidities not mentioned, dengue and other communicable diseases, studies restricted to pregnancy and children, review articles, case series and case reports, other languages than English, French, Portuguese or Spanish, and full text not available.

An Excel spread sheet was developed to extract data from studies. Information collected were based on the PICO approach and included: general information about the article, studies designs and hypothesis, participants’ features, outcome data, results and main findings. The studies were analysed using a comparative approach of the extracted details. Because of the paucity and relatively low level of evidence of the identified studies, no quality assessment has been performed for further exclusion of articles, however the quality of the studies has been discussed in the discussion section.

## Results

### Study selection process

**Fig 1:**
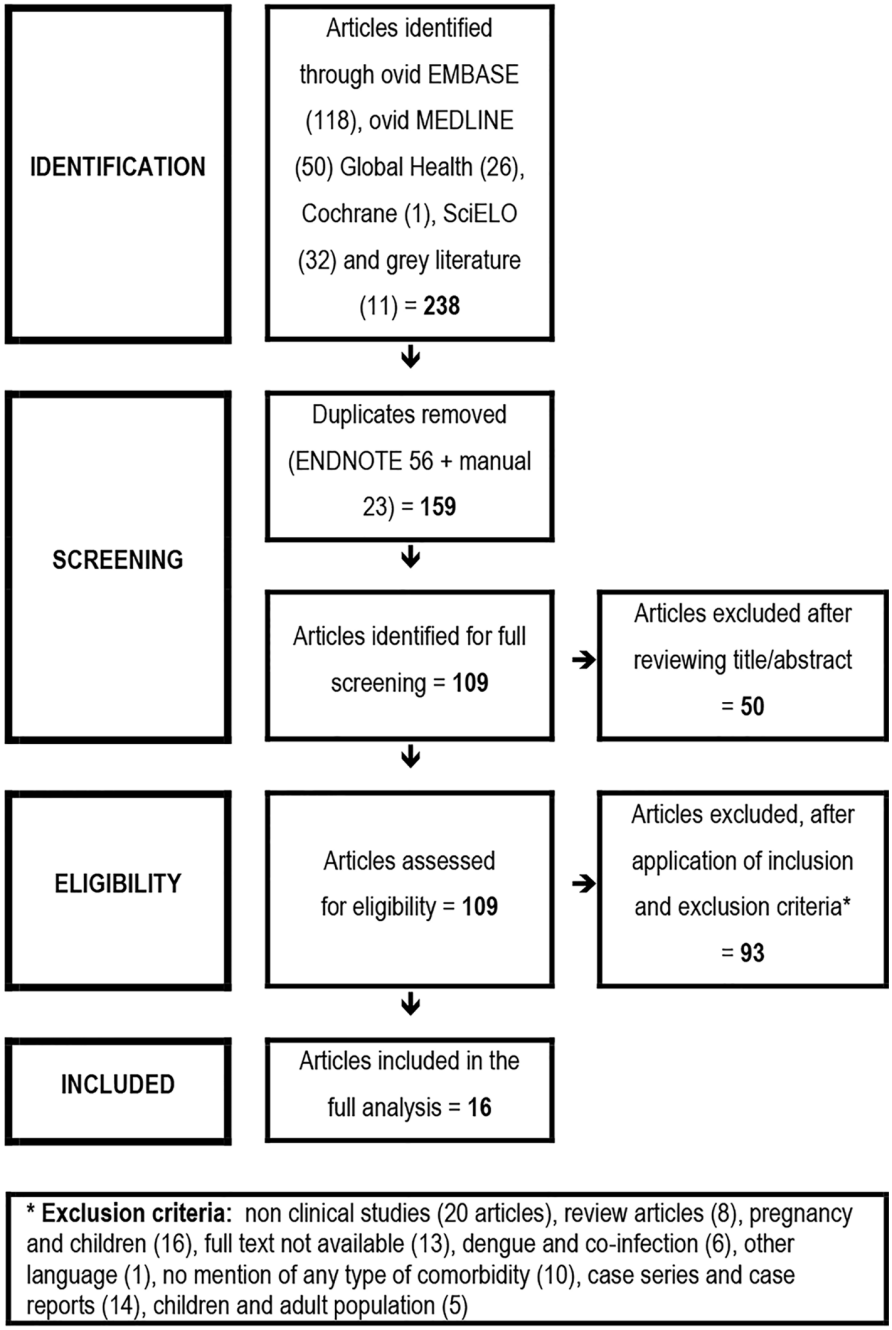
Flowchart of the study selection process.

A total of 238 potentially relevant articles were initially identified, 129 were excluded after screening the title and abstract. 109 articles were retrieved as full articles. A further 93 articles were excluded after applying all inclusion and exclusion criteria. 16 articles were included in the analysis (Fig 1 for the flowchart of the selection process; Table 2 provides an overview of the studies). A meta-analysis was not performed, due to the heterogeneity of outcome measures.

**Table 2:** Included studies.

First author, year, country, and period of study	Main objectives	Sample size	Criteria for inclusion of cases	Criteria for exclusion of cases	Main results				
**Case control studies**									
Teixeira MG, 2015, Brazil, 2009–2012 (27)	To investigate the hypothesis that some specific comorbidities increase the likelihood of a dengue fever case progressing to dengue haemorrhagic fever	N = 1806 patients	Patients admitted in the hospitals with signs and symptoms of dengue fever were followed up until a final diagnosis. Those who progressed to DHF/DSS were classified as cases and those who did not progress for DHF were classified as controls.	Not mentioned	Hypertension (OR = 1.6; 95CI 1.1–2.1) and skin allergy (OR = 1.8; 95CI 1.1–3.2) were associated with DHF (adjusted for ethnic and social variables)				
		Cases: dengue haemorrhagic fever patients (N = 490)	Case definition: Patients with dengue fever who progressed to DHF according to the WHO 1997 criteria		Self-reported skin allergies (OR = 2.1; 95CI 1.1–4.1), and hypertension (on anti-hypertensive drugs) (OR = 1.4; 95CI 1.1–2.0) were associated with DHF. Also, when patients were not taking antihypertensive drugs, there was also an association (OR = 1.8; 95CI 1.1–3.2)				
		Controls: (N = 1316)	Control definition: Patients, from the same hospital as cases, with signs and symptoms of DF and positive specific laboratory diagnosis for dengue who did not progress to DHF.						
Karunakan, India, 2013, 2005–2008 (17)	To identify risk factors of mortality in patients with a confirmed diagnosis of DF during hospital stay	N = 50 patients (10 cases and 40 controls)	Cases: patients admitted to the hospital with probable DF and confirmed laboratory diagnosis and who died during hospital stay.	Not mentioned	Comorbidities	Cases (n = 10)	Controls (n = 241)	*OR (95CI)*	p-value
			Controls: patients with laboratory confirmation of DF who recovered from illness and then discharged		Diabetes	4 (40)	1 (2, 5)	26 (2.5–273.7)	0.004
					Hypertension	7 (70)	2 (5)	44.3 (6.2–315.5)	0.000
Mahmood S, 2013, Pakistan, 2011 (24)	To evaluate the relationship between comorbidities (diabetes mellitus, cardiovascular diseases, bronchial asthma, tuberculosis, and chronic liver disease) in patients with DF and progression to DHF	N = 373 patients	Cases: Male/female patients aged between 15–65 years old and with a diagnosis of DHF according to 1997 WHO classification	Not mentioned	Comorbidities	Cases (n = 132)	Controls (n = 241)	*aOR (95CI)*	p-value
		Cases: 132 DHF/DSS	Controls: patients from the same hospitals with a positive anti-dengue IgG and matched with cases for age/sex		Diabetes mellitus	57 (43, 2)	104 (41.8)	1.26 (0.78–2.03)	0.34
		Controls: 241 patients			Hypertension	67 (50.8)	135 (54.2)	0.93 (0.57–1.49)	0.76
					Ischaemic heart disease	26 (19.7)	44 (17.7)	1.52 (0.85–2.73)	0.15
					Bronchial asthma	14 (10.6)	23 (9.2)	1.34 (0.62–2, 88)	0.44
					Chronic liver disease	12 (9.1)	27 (10.8)	Not mentioned	
					Pulmonary tuberculosis	10 (7.6)	13 (5.2)	1.41 (0.57–3.43)	0.44
Thein T-L, 2013, Singapore, 2004–2008 (28)	To identify demographic, clinical and laboratory risk factors related to death in adult patients with dengue fever	N = 108 patients admitted to the hospital with a diagnosis of dengue fever and later died due to complications	Cases: patients with laboratory confirmed diagnosis of dengue who died on a cohort of patients admitted to hospital (N: 28)	Not mentioned	Comorbidities	Non fatal (n = 80)	Fatal (n = 28)	OR (95CI)	p-value
			Controls: patients with laboratory confirmed diagnosis of dengue who did not die in a cohort of patients admitted to hospital (N: 80)		Diabetes mellitus	17(21.2)	9 (39.1)		0.078
					Hypertension	35 (43.8)	12 (50.0)		0.821
					Heart failure	2 (2.5)	2 (11.1)		0.176
					Hyperlipidemia	16 (20.0)	3 (16.7)		0.503
					Cardiac disorder	7 (8.8)	10 (47.6)	10.643 (2.274–49.821)	0.003
					Renal disorder	2 (2.5)	8 (40)	21.176 (2.63–170.54)	0.004
Lee CC, 2013, Taiwan, January–December 2007 (19)	To compare demographic and clinical features of elderly and young adult patients with dengue fever	N = 193 patients Cases: elderly patients (N = 31)	Patients that visited the Emergency Room Department and had dengue fever suspected by the physician	Cases / Controls: patients with negative serology for dengue fever (exclusion of 73 individuals)	The overall mortality for the dengue cases was 0.5%.				
		Controls: young adults (N = 162)	Cases: elderly adults (>65 years older)		However, mortality for elderly cases was higher (p<0.0001).				
			Controls: young adults (<65yo)		When comparing the two groups, the elderly presented with a higher proportion of comorbidities:				
					1 comorbiditiy <0.01				
					> 2 comorbidities 0.001				
					Hypertension 0.009				
					Diabetes mellitus 0.01				
					Coronary artery disease 0.05				
					Chronic renal insufficiency 0.59				
					COPD 0.51				
					Liver cirrhosis 0.41				
					Malignancy 0.01				
					Old stroke 0.07				
Pang J, 2012, Singapore, January 2006 –December 2008 (25)	To evaluate risk factors (demographic data and comorbidities) for DHF in adults during two dengue outbreaks	N = 2285 patients	Hospital-based cases / controls	Not mentioned	OR comparing cases of DHF to controls (DF)				
		Cases: DHF (N = 818)	Cases: patients with DHF			OR crude (95CI)		OR adjusted (95CI)	
		Controls: DF (N = 1467)	Controls: patients with DF			2006 outbreak	2007/2008 outbreak	2006 outbreak	2007/2008 outbreak
					Hypertension	1.84 (0.74–4.54)	1.41 (1.02–1.94)	0.97 (0.31–3.00)	1.06 (0.7–1.6)
					Diabetes mellitus	0.62 (0.13–3.02)	1.89 (1.21–2.94)	0.34 (0.06–1.89)	1.78 (1.06–2.97)
					Hyperlipidaemia	0.97 (0.29–3.2)	1.24 (0.87–1.76)	0.54 (0.15–1.96)	0.79 (0.5–1.26)
					Asthma	0.57 (0.19–1.75)	0.92 (0.59–1.43)	0.51 (0.16–1.62)	0.86 (0.55–1.35)
Figueiredo MAA, 2010, Brazil, 2003–2005 (16)	To evaluate whether patients with comorbidities were at higher risk of developing DHF rather than DF	N = 1345 patients	Cases: Cases of DHF included in the national surveillance system and that met criteria for DHF after chart revision by physician; laboratorial confirmation	Cases: patients with diagnosis of severe dengue but no criteria for DHF and insufficient information	OR comparing cases of DHF to controls (DF)				
		Cases: DHF (N = 170)	Controls: individuals from the same neighbourhood as the case and that had had DF in the same year as the case, matched by age/sex	Controls: not mentioned		OR crude (95CI)		OR adjusted (95CI)	
		Controls: healthy individuals with a history of DF in the same year (N = 1175)			Hypertension	0.9 (0.5–1.62)		0.93 (0.51–1.70)	
					Diabetes	2.46 (1.03–5.87)		2.75 (1.12–6.73)	
					Allergy	1.59 (1.11–2.28)		1.29 (0.87–1.89)	
					Asthma	0.93 (0.46–1.89)		0.87 (0.41–1.84)	
**Retrospective Cohort studies**									
Saqib Man, 2014, Pakistan, 2011 (26)	To analyse the initial presentations of dengue fever cases and to estimate the frequency of comorbidities in these patients	N = 556 patients admitted to two hospitals with confirmed laboratorial diagnosis of dengue fever. Of these, 48 patients died and causes of death were evaluated through verbal autopsies	Retrospective analysis of records of patients with dengue fever admitted at the hospital. Application of verbal autopsy questionnaire on relatives of 48 deceased patients	Not mentioned	29/40 (60%) of the deceased cases had a diagnosis of comorbidity (diabetes mellitus, high blood pressure, asthma, HIV or viral hepatitis B/C). 20 deceased patients had hypertension either alone or along with any other illness and majority of them suffered from DSS. Similarly diabetes and hepatitis B or C were also major risks for developing DSS				
Chamnanchanunt S, 2012, Thailand, 2006–2009 (15)	To identify risk factors for clinically significant bleeding in patients with DF	N = 270 patients (based on sample estimates): Type I bleeding (N = 97), Type II/III bleeding (N = 180)	Patients >15 years old and laboratorial confirmation of dengue virus infection	Patients with co-infections and other bleeding events or medical conditions that predispose bleeding (liver disease, haematological disease, antiplatelet and anticoagulation medication)	Risk factors contributing to significant bleeding		p-value		
					Respiratory illness		0.203		
					Hypertension		0.954		
					Metabolic disorder		0.663		
					Gastrointestinal illness		0.87		
Lee IK, 2012, Taiwan, June–December, 2002 (21)	To compare clinical features and laboratory parameters of a pool of patients with DHF who died	N = 309 cases of DHF: Fatal cases (N = 10), Survival cases (N = 299)	Patients with clinical and laboratorial diagnosis confirmation of DF on that period	No mention of exclusion criteria	Risk factors contributing to death		p-value		
				There were initially 714 patients with the diagnosis of “dengue illness”	Diabetes mellitus		0.693		
					Hypertension		0.495		
					COPD		0.99		
					Previous stroke		0.544		
					Chronic kidney disease		0.07		
					Parkinsonism		0.094		
					Solid tumor		0.124		
Low JG, 2011, Singapore, April 2005 –August 2010 (23)	To examine clinical features of DF in different age groups in the context of early clinical diagnosis	N = 2129 patients	Patients aged > 18 years old and with acute history of fever, reporting at specific health facilities, patients were followed up, laboratorial confirmation of cases	Not mentioned	The frequency of comorbidities in cases of dengue increases with age				
		Dengue (N = 250)			Hospitalisation increases with age				
		OFI (N = 1879, subset of 228 patients tested (+) influenza A and B							
Lye DC, 2010, Singapore, 2004 (12)	To compare dengue patients aged ≥60 years old with younger adults patients in terms of comorbidities, disease severity and outcome	N = 1971 patients	Patients admitted during the year of 2004 who fulfilled the WHO criteria for acute dengue and positive laboratorial test; patients classified into DF, DHF, DSS;	Not mentioned	Comparing the two age groups of dengue patients, the median length of hospitalisation was longer in the elderly (p = 0.1), with no difference for intensive care unit admission (p = 1) and the number of deaths (p = 1)				
		Older patients (> 60 years old) (N = 66)			Comparing the risk factors between the two age groups		p-palue		
		Younger patients (< 60 years old) (N = 1905)			Diabetes mellitus		0.0001		
					Hypertension		0.0001		
					Ischemic heart disease		0.0001		
					Hyperlipidemia		0.0001		
Lee IK, 2009, Taiwan, June–December, 2002 (21)	To study the clinical characteristics and outcomes of DHF patients with acute renal failure (ARF) and to identify risk factors for development of ARF in patients with DHF	N = 304 patients:	Patients >18 years old with laboratory confirmation of DF	Not mentioned	Case fatality was higher in cases of DHF with ARF (p<0.0001)				
		DHF and ARF (N = 10)			Comparing the risk factors contributing to DHF with ARF		p-value		
		DHF and non ARF (N = 294)			Diabetes mellitus		0.693		
					Hypertension		0.071		
					Previous stroke		0.005		
					Chronic renal disease		0.046		
Kuo MC, 2008, Taiwan, January 2002 –January 2003 (18)	To evaluate whether dengue viral infections in patients with renal failure have different clinical presentations and disease outcome	N = 519 patients with dengue fever: Renal failure (N = 21), Non renal failure (N = 498)	Patients with clinical and laboratory diagnosis of DF	Cases without clinical confirmation or detailed clinical history	Case fatality was higher with dengue and renal failure (p<0.0001)				
				28 of the 549 were excluded because of incomplete information (clinical charts and baseline serum creatinine)	Comparing the risk factors contributing to a different clinical presentation and outcome between dengue cases with or without renal failure		p-value		
					Previous renal failure		<0.01		
					Cancer		0.24		
					Diabetes		0.03		
					Hypertension		<0.01		
					Cardiovascular disease		0.13		
					Pulmonary diseases		1.0		
					Rheumatological diseases		<0.01		
					Gastrointestinal disease		0.8		
Lee IK, 2008, Taiwan, June–December 2002 (20)	To understand the clinical characteristics of DHF in the elderly and non—elderly adults and to identify risk factors for fatality in the elderly population	N = 307 patients:	Elderly patients (≥ 65 years old) with confirmed laboratory diagnosis of DF	Not mentioned	Case fatality rate was higher (p = 0.049) and length of hospital stay was longer (p = 0.006) in the elderly group				
		Elderly patients (N = 66)			Comparing the risk factors in both groups		p-value		
		Non elderly patients (N = 241)			Diabetes mellitus		0.058		
		Subgroup analysis of the elderly group: fatal cases (N = 5) non-fatal cases (N = 61)			Hypertension		0.001		
					Previous stroke		0.001		
					COPD		0.001		
					Chronic renal disease		0.001		
					Corticosteroid use		0.001		
					Malignancy		0.068		
					Heart disease		0.293		
Wang CC, 2007, Taiwan, June–December 2002 (29)	To evaluate the clinical course and outcome of dengue patients with acute respiratory failure and to identify risk factors	N = 606	Patients with DF (clinical and laboratory confirmation)	Exclusion of 55 patients aged <18yo	The length of hospital stay was longer in the group of cases with acute respiratory failure				
		Acute respiratory failure, (N = 11)			Comparing the risk factors in both groups		p-value		
		No acute respiratory failure (N = 595)	aged >18 years old		Diabetes mellitus		0.305		
					Hypertension		0.036		
					COPD		0.039		
					Stroke		0.049		
					End stage renal disease		0.010		
					Malignancy		0.257		

### Descriptive analysis of the studies

#### Study designs, settings and objectives

Sixteen articles, describing studies conducted from 2002 to 2012 met the eligibility criteria [12, 15, 16, 17, 18, 19, 20, 21, 22, 23, 24, 25, 26, 27, 28, 29]. All studies included in the analysis were observational studies: nine retrospective cohort studies [12, 15, 18, 20, 21, 22, 23, 26, 29] and seven case-control studies [16, 17, 19, 24, 25, 27, 28]. Geographically, six studies were conducted in Taiwan [18, 19, 20, 21, 22, 29], four in Singapore [12, 23, 25, 28], two each in Brazil and Pakistan [16, 24, 26, 27] and one in India and Thailand [15, 17].

The studies had a wide range of objectives. Eight studies analysed hypertension, diabetes mellitus and allergies as risk factors [15, 16, 17, 24, 25, 26, 27, 28]. Four studies had as main objectives the study of old age as a risk factor for the development of the severe forms of dengue [12, 19, 20, 23] and two studies focused on risk factors for renal failure [18, 21]. One study assessed risk factors for mortality in patients with DHF [22]. One study focused on risk factors and comorbidities linked to the development of acute respiratory failure in dengue cases [29].

In order to report on the outcome measures DF, DHF, DSS and SD, in this systematic review “severe forms of dengue” is used to describe dengue severity in more general terms; however, for the specific reporting of the studies, the case definitions and classifications as described in the studies are maintained.

#### Sample size of included studies

The studies involved a total of 13,139 adult patients, ranging from 50 to 2,285 patients, aged 15 years or more. All seven case-control studies described clear inclusion criteria for both cases and controls. Exclusion criteria were reported for only two of the studies. All nine retrospective cohort studies reported inclusion criteria; common to all studies was laboratory (serology or molecular biology) confirmation of DENV infection. Of the cohort studies, only three studies reported exclusion criteria [15, 18, 29].

#### Exposure, outcome and measures of association

All studies measured different risk factors and also considered different outcomes.

For the case control studies, two studies [16, 25] considered severity of dengue (DHF) as the primary outcome, assessing comorbidities as risk factors. The comorbidities evaluated included hypertension, diabetes mellitus, hyperlipidaemia, asthma and allergy. In the study by Pang et al [25], diabetes and diabetes and hypertension were analysed in relation to DHF. Figueiredo et al [16] analysed diabetes and allergy.

Lee et al [19], assessed the influence of age on the length of hospital stay and mortality rate on day 14 from the initial visit to the Emergency Department. The authors compared also comorbidities between the two age groups.

For the retrospective cohort studies, five of eight studies evaluated case fatality rates / mortality rates / deaths as outcome measures in patients with dengue [12, 18, 20, 21, 22]. Evaluation of comorbidities included cardiovascular disease and stroke, respiratory illnesses such as chronic obstructive pulmonary disease (COPD), renal disease, cancer, diabetes, Parkinsonism, but also hypertension and hyperlipidaemia (see Table 2 for the full list).

### Summary of the main results of the studies

#### Case control studies (Table 2)

Pang et al [25] conducted a case control study in Singapore to evaluate the role of comorbidities in the development of DHF. The findings showed that diabetes and hypertension were associated with an elevated risk of DHF in the 2007/2008 outbreak: crude Odds Ratio (OR) 1.41 (1.02–1.94) for hypertension and 1.89 (1.21–2.94) for diabetes mellitus. However, results in other years and also the adjusted ORs did not confirm these results. Figueiredo et al [16] found in a study in Brazil an association between DHF and diabetes and allergies (crude ORs: 2.46 (1.03–5.87) and 1.59 (1.11–2.28) respectively). Similar results were found by Teixeira et al [27], who found an association between hypertension (adjusted OR 1.6; 95% Confidence Interval (95CI) 1.1–2.1), skin allergy (adjusted OR 1.8; 95CI 1.1–3.2) and progression to DHF. In addition to that, findings by Karunakan et al [17] showed that diabetes (crude OR 26 (2.5–273.7) and hypertension (crude OR 44.3 (6.2–315.5) increased the risk of mortality amongst DHF cases admitted to hospital. Conversely, despite its relevant sample size, Mahmood et al [24] did not find an association between comorbidities and development of DHF.

Lee CC et al [19] compared elderly versus younger patients, with DHF as the outcome measure. The authors concluded that the former were more prone to present DHF, higher disease severity and more comorbidities than the latter. Older age, as a risk factor, was related to longer length of hospital stay. Thein T-L et al [28] found that cardiac and renal disorder were related to fatality in DHF cases admitted to a hospital in Singapore.

#### Retrospective cohort studies (Table 2)

These studies presented several exposures and outcomes. Of the nine retrospective cohort studies, six studies [12, 18, 20, 21, 23, 26] showed findings in favour of comorbidities as risk factors for the severe forms of dengue whereas three [15, 22, 29] found no relationship.

Chamnanchanunt et al [15] compared types of bleeding (type I, clinically significant bleeding *versus* type II/III bleeding) in relation to comorbidities. None of the analysed comorbidities appeared to be related to the severity of bleeding (respiratory illness, hypertension, metabolic disorder, gastrointestinal illness).

Two studies evaluated renal impairment in the development of severe forms of dengue. Kuo et al [18] studied the impact of chronic renal failure on dengue severity. Their findings showed that patients with dengue and severe renal function impairment had a higher risk of mortality and higher possibility of having DHF/DSS compared to the control group (p<0.0001). The cases with renal failure also presented more often with comorbidities, especially hypertension, diabetes and rheumatological conditions. Lee et al [21], evaluating retrospectively a cohort of patients with dengue who developed acute renal failure, concluded that there was good evidence that age, male sex, previous stroke, chronic renal disease and fatality rate were associated with DHF and acute renal failure, but not hypertension and diabetes. The case fatality rate of dengue was high once acute renal failure developed.

Two studies [12, 23], assessed the length of hospitalisation of dengue infected patients, with opposite findings in relation to the role of comorbidities. Low et al [23] concluded that older adults less frequently reported clinical symptoms of dengue than younger adults, delaying the diagnosis. However, hospitalisation increased with age. Lye et al [12] observed that patients aged ≥ 60 years presented with more comorbidities (hypertension, diabetes mellitus, ischaemic heart disease and hyperlipidaemia) than patients aged < 60 years. When comparing the two groups for intensive care unit admission and death, no differences were noted.

Lee et al [20] studied risk factors for dengue death in the elderly population. The authors concluded that elderly patients had a higher mortality rate of dengue compared to younger patients and that DSS is an independent risk factor for death (p = 0.006) in elderly patients with DHF. In this study diabetes mellitus was not a risk factor for DHF, but hypertension, stroke, COPD, chronic renal disease and corticosteroid use.

A study set in Taiwan [22] evaluated clinical and laboratory risk factors of patients who developed DHF and subsequently died and there was no statistical difference in the frequency of comorbidities with patients who survived.

Wang et al [29] analysed cases of dengue who developed acute respiratory failure. This group of cases presented more often with hypertension, COPD, stroke and renal disease. Mortality rate in this cohort was very high with 73%.

A study by Saqib et al [26] performed verbal autopsies on deceased cases of DF admitted to tertiary hospitals in Pakistan. The authors concluded that 29/40 (60%) of the deceased cases had a diagnosis of comorbidity (diabetes mellitus, high blood pressure, asthma, HIV or viral hepatitis B/C). About 20 deceased patients had hypertension either alone or along with any other illness and the majority of them suffered from DSS. Similarly diabetes and hepatitis B or C were also another major risks for developing DSS.

## Discussion

The studies retrieved in this review measured different outcomes (DHF, DSS and SD—with different definitions for severity), making results challenging to compare. Since 2009, WHO has adopted a new dengue case classification (dengue and severe dengue: D/SD) [3] to improve clinical management. In this systematic review, despite the fact that all studies were conducted and published after 2002, only Low JG et al [23] designed their study based on the most recent classification of dengue. One of the implications is that future studies should use similar endpoint measures, to ensure an improved comparability.

Similarly, when assessing comorbidities, the studies showed that many different comorbidities have been analysed. An agreed set of comorbidities should be analysed in future studies. No clear analysis of the influence of comorbidities on the development of severe dengue can be shown from this analysis, but recurrently 1) cardiovascular disease 2) stroke, 3) diabetes, 4) respiratory disease and 5) renal disease were associated with the severe forms of dengue, as well as old age. A recent systematic literature review and meta-analysis by Htun NS et al [30] about the causal effect of diabetes on severe clinical presentations of dengue fever was inconclusive; however the authors suggest that glycaemic triage in patients with suspicion of DF should be performed.

The variability of study designs is another issue, particularly the source of study population (patients admitted to hospitals). Ideally, studies related to comorbidities should also include primary care facilities, where the vast majority of dengue cases are seen. Only case-control and retrospective studies were identified in the systematic review, furthermore, often with very small study populations, with the inherent limitations for the interpretation of findings [31].

These features also identify the need for bigger and especially prospective studies, assessing the role of comorbidities in dengue severity with standardised protocols.

This systematic literature review has its limitations, since the presence of comorbidities in individuals with dengue is a subject underexplored in the scientific literature. Furthermore, most of the clinical data are not recorded as risk factors and thus retrieving this information becomes difficult in databases. The studies retrieved were heterogeneous for exposures and outcomes, making it difficult to extrapolate results. The problems were addressed by including all studies related to the research question in the analysis, and not excluding because of lack of quality—as a result, the analysis permitted only for a descriptive analysis.

### Conclusion

The results presented highlight that comorbidities might influence the development of severe forms of dengue. Further research including standardised prospective cohort studies in regions with high prevalence of DENV infection would contribute to a better understanding of the relevance of comorbidities in severe dengue.

## Supporting Information

S1 Checklist PRISMA Checklist. (DOCX)
